# Divergent responses of adiponectin and leptin to exercise in women with overweight or obesity: a systematic review and meta-regression

**DOI:** 10.3389/fphys.2026.1810408

**Published:** 2026-07-15

**Authors:** Xiufeng Yuan, Chunyu Bao, Xuequan Feng

**Affiliations:** 1 Tianjin University of Sport, Tianjin, China; 2 Tianjin Sports Injury and Rehabilitation Virtual Simulation Teaching Center, Tianjin, China; 3 The Neurosurgical Department, Tianjin first Central Hospital, Tianjin, China

**Keywords:** adiponectin, dose-response, exercise volume, leptin, meta-regression, obesity

## Abstract

**Objective:**

We examined how the volume of exercise relates to circulating adiponectin and leptin in women with overweight or obesity, asking whether each hormone responds in proportion to the amount of exercise accumulated or instead changes only once a sufficient stimulus has been reached.

**Methods:**

We searched five databases (Web of Science, PubMed, Cochrane Library, MEDLINE, and Embase) for randomized controlled trials (RCTs) appearing through January 2026 (PROSPERO: CRD420261438448) and pooled standardized mean differences (SMDs) under random-effects models. Weekly exercise volume (minutes per week) was modelled as a continuous moderator in univariate meta-regression, and moderate-intensity continuous training (MICT) was compared with high-intensity interval training (HIIT).

**Results:**

Thirty-four RCTs (N = 1,987) were analyzed. Exercise raised adiponectin (SMD = 0.60; P < 0.05) and lowered leptin (SMD = -0.70; P < 0.01), but neither response scaled with the weekly volume of exercise. In univariate meta-regression the slope for weekly volume was non-significant for both adiponectin (slope = -0.0039, P = 0.331) and leptin (slope = 0.0031, P = 0.394), consistent with a threshold effect rather than a gradual, volume-dependent change. Where the hormones diverged was in intensity rather than volume: the pooled adiponectin effect was numerically larger for MICT than for HIIT, although the subgroup difference was not statistically significant, whereas the leptin reduction was similar across intensities. Because energy intake and expenditure were not reported consistently, the contribution of energy balance to the leptin response could not be tested directly.

**Conclusion:**

Adiponectin and leptin both improve with exercise, but neither response scales with the weekly volume of training. Adiponectin rose more with moderate-intensity continuous training (MICT) than with HIIT, whereas the leptin reduction was similar across intensities; in both cases the change appeared once an adequate stimulus was delivered rather than accumulating with extra weekly minutes, with energy balance a likely but untested contributor to the leptin response. Prescription can therefore be tailored to the target: moderate-intensity continuous aerobic training when the aim is to raise adiponectin, and either MICT or shorter HIIT when the aim is to lower leptin, provided that energy balance is also managed.

**Systematic review registration:**

https://www.crd.york.ac.uk/PROSPERO/view/, identifier CRD420261438448.

## 1 Introduction

Obesity in women is becoming more common, and it is no longer seen as a simple mismatch between energy taken in and energy spent. It is better described as a multifactorial disorder in which adipose tissue itself becomes structurally and functionally abnormal, a process shaped in women by sex-specific patterns of fat distribution (Karastergiou et al., 2012). When energy intake chronically exceeds expenditure, subcutaneous depots reach the limit of their storage capacity and lipid spills over into the visceral compartment (Virtue and Vidal-Puig, 2010). This visceral accumulation marks a turning point, as adipose tissue changes from an inert store of energy into an active, pro-inflammatory endocrine organ (Coelho et al., 2013). With this change the tissue alters its output of adipokines, the signaling peptides through which fat communicates with the rest of the body. The balance between adiponectin and leptin is especially relevant, because its disruption helps sustain the low-grade inflammation that underlies insulin resistance, endothelial dysfunction, and raised cardiovascular risk in women with overweight or obesity (Ouchi et al., 2011; Blüher, 2019).

The two adipokines most often studied in this setting have contrasting roles. Adiponectin, the most abundant of them, improves insulin sensitivity and dampens inflammation: in skeletal muscle it activates AMP-activated protein kinase (AMPK) to promote fatty-acid oxidation, and in the endothelium it suppresses nuclear factor-κB (NF-κB) signaling and so limits vascular inflammation (Yamauchi et al., 2002; Turer and Scherer, 2012). Paradoxically, adiponectin production falls in obesity, so this protection is weakened just when it is most needed. Leptin is more complicated. Its concentration rises with fat mass, yet many people with obesity develop selective leptin resistance: the brain no longer registers leptin’s satiety signal, while the sympathetic nervous system stays responsive to its stimulatory effects and so contributes to hypertension and metabolic inflexibility (Myers et al., 2010). Women with obesity thus carry two linked problems at once, weakened insulin sensitization from low adiponectin and disordered control of energy balance from leptin resistance.

Exercise is a mainstay of non-drug treatment for this endocrine disturbance. Its anti-inflammatory effects are well established, and regular training lowers visceral fat and shifts the circulating mix of myokines and adipokines in a healthier direction (Gleeson et al., 2011). Turning these biological findings into concrete clinical advice has proved harder, mainly because the available trials differ widely and give inconsistent estimates of how dose relates to benefit (Batacan et al., 2017). One question remains open: does a healthier adipokine profile build up gradually with more exercise, so that each additional minute adds benefit, or does it emerge only once a certain intensity or duration has been reached?

Existing meta-analyses have tended to blur these distinctions. Most pool adipokines together as a single class and assume they all react to exercise in the same way (Reseland et al., 2001). Such averaging can mask quite different behavior. Adiponectin, for instance, may respond to the total amount of aerobic exercise, whereas leptin tends to move little unless training is accompanied by meaningful weight loss. The part played by intensity is also unsettled, particularly the contrast between moderate-intensity continuous training (MICT) and high-intensity interval training (HIIT). HIIT is time-efficient (Gibala et al., 2012), but whether its shorter metabolic stimulus can ease chronic low-grade inflammation as effectively as the longer sessions of MICT is not yet clear. As long as adipokines that probably behave differently are pooled together, and intensities are not compared head to head, firm exercise recommendations will remain out of reach.

Three recent reviews illustrate these gaps (Fedewa et al., 2018) conducted the most comprehensive meta-analysis of exercise and leptin to date, covering 40 RCTs, but conflated aerobic with resistance training, reported weekly volume only as a binary categorical variable (≥150 versus <150 min/week) rather than as a continuous moderator, and did not analyze adiponectin separately; nor did that review restrict its sample to women or distinguish between MICT and HIIT (Batacan et al., 2017) focused on HIIT and cardiometabolic outcomes across 39 studies but used mixed-sex samples, reported adipokines as part of a broad inflammatory cluster rather than analyzing adiponectin and leptin individually, and did not estimate a dose-response slope or compare HIIT directly with MICT for either hormone (Wang et al., 2024) compared HIIT with MICT in adolescents but combined adipokine and cardiovascular endpoints into a single composite, did not model weekly volume as a continuous variable, and restricted enrolment to youth, leaving the question open for adult women with overweight or obesity. Taken together, these three reviews — and, to our knowledge, every earlier meta-analysis — share three methodological gaps: adiponectin and leptin are reported as a single combined adipokine response, weekly training time is rarely modelled as a continuous moderator in meta-regression, and MICT and HIIT are seldom compared hormone by hormone in female-only samples. The present review was designed to close each of these gaps in women with overweight or obesity.

We therefore set out two predictions in advance. The first concerns adiponectin: because its gene expression depends on local tissue oxygenation and on PPARγ-driven transcription, both of which need sustained metabolic input to change, we expected adiponectin to rise with the total volume of aerobic exercise, more steeply in protocols built on sustained duration (MICT) than on brief, intense efforts (HIIT). The second concerns leptin: because it mirrors fat mass and short-term energy availability rather than slow tissue remodeling, we expected its reduction to behave like a threshold effect, appearing once a sufficient stimulus is delivered but changing little with extra weekly minutes and largely the same whether delivered as MICT or HIIT. These two predictions set the framework for the meta-regression and subgroup analyses and constrain how we interpret the results.

The aim of this study was to test these predictions through a systematic review and meta-regression of randomized controlled trials in women with overweight or obesity, and to use the results to guide more targeted exercise advice for this group.

## 2 Methods

This systematic review was prepared following the PRISMA 2020 reporting guidance (Page et al., 2021). The protocol was registered in PROSPERO (CRD420261304139) prior to data extraction; because the search was completed in January 2026 and registration was finalized in February 2026, registration was retrospective to the search but prospective to all data extraction, quality assessment, and analysis steps.

### 2.1 Literature search strategy

Five electronic databases — PubMed, Web of Science, Embase, MEDLINE, and the Cochrane Library — were searched from their earliest records to January 2026 for randomized controlled trials (RCTs) of exercise interventions and circulating adipokines in women with overweight or obesity. The search followed the PICOS framework: the population (P) was women with overweight or obesity, both pre- and postmenopausal; the intervention (I) was structured exercise, whether aerobic, resistance, high-intensity interval training (HIIT), or combined; the comparator (C) was non-exercise, sedentary, or minimal-activity control; the outcomes (O) were the adipose-derived hormones adiponectin and leptin; and the design (S) was the randomized controlled trial.

Search strings combined Medical Subject Headings (MeSH) and free-text terms joined by Boolean operators, for example (“Obesity” OR “Overweight” OR “Body Mass Index” OR “Adiposity”) AND (“Exercise” OR “Physical Activity” OR “Aerobic Training” OR “Resistance Training” OR “Interval Training”) AND (“Adiponectin” OR “Leptin” OR “Adipokines”). No language or date limits were applied. We also hand-searched the reference lists of the included studies and related reviews for trials that the databases had missed. Two reviewers ran the searches independently and resolved any differences by discussion. The full Cochrane Library search string is shown in **Supplementary Figure 1**.

### 2.2 Eligibility criteria

Inclusion and exclusion criteria were defined from the same PICOS framework (Page et al., 2021). The full criteria are listed in **Supplementary Table 1**.

### 2.3 Study selection and data extraction

Study selection and data extraction were carried out independently by two investigators, following the PRISMA guidance (Page et al., 2021), and a third senior reviewer settled any disagreement. Screening ran in two stages: titles and abstracts were checked first to drop clearly irrelevant records, after which the full texts of the remaining studies were assessed against the inclusion criteria, as shown in **Supplementary Figure 2**. When data were missing or unclear, corresponding authors were emailed, and a study was excluded if no reply arrived after two attempts. A piloted extraction form recorded bibliographic details (author, year, country), participant characteristics (sample size, age, menopausal status, baseline BMI, body-fat percentage), and the intervention coded by the FITT principle (Thompson et al., 2013) (frequency, intensity, session duration, type, and total length), together with baseline and post-intervention means and standard deviations for Adiponectin and Leptin.

### 2.4 Risk of bias and methodological quality assessment

Two reviewers independently judged methodological quality and risk of bias for the 34 included trials, using the Cochrane Handbook (Higgins et al., 2011). A summary of these judgments appears in **Supplementary Figure 3**, and the study-by-study assessments in **Supplementary Figure 4**. Within each of the tool’s seven domains — sequence generation, allocation concealment, blinding of participants and personnel, blinding of outcome assessment, incomplete data, selective reporting, and other bias — every trial was graded low, unclear, or high risk. We then tallied the share of trials at each risk level per domain and rolled the domain grades up into a single overall judgment for each study.

### 2.5 Data synthesis and statistical analysis

Analyses were carried out in R (version 4.3.1), using the metafor package for computation and ggplot2 for figures (Balduzzi et al., 2019). We expressed each effect as a standardized mean difference (SMD) with 95% confidence intervals (CIs), a metric that lets results obtained with different assay kits be combined. Effects were computed from pre-to-post change scores; where the SD of the change was not reported, we recovered it from the baseline and post-intervention SDs by setting the within-subject correlation to r = 0.5, the value recommended in the Cochrane Handbook (Cumpston et al., 2022).

Anticipating clinical and methodological diversity across the trials, we pooled effects with a random-effects model estimated by restricted maximum likelihood (REML). Between-study inconsistency was gauged from the I² statistic (Higgins and Thompson, 2002); following convention, values near 25, 50, and 75% were read as limited, moderate, and substantial inconsistency between trials. To trace this inconsistency, weekly exercise volume was entered as a continuous moderator in univariate meta-regression, and the pooled effects of the MICT and HIIT subgroups were contrasted.

We screened both outcomes for small-study effects and publication bias by examining funnel plots alongside Egger’s regression test (Egger et al., 1997). The influence of individual trials was examined using Cook’s distance, and studies exceeding the conventional threshold of 4/n were flagged as potentially influential (**Supplementary Figure 5**). All tests were two-sided, and we treated P < 0.05 as significant.

## 3 Results

### 3.1 Characteristics of included studies

The review included 34 RCTs and 1,987 women with overweight or obesity. Trial size ranged from 16 to 215 participants, mean age from 13.1 to 66.78 years, and mean baseline BMI from 23.3 to 37 kg/m². The interventions spanned aerobic, resistance, high-intensity interval, and concurrent training, and varied widely in dose: session length had a median of 53 minutes (range 30–140), frequency was 2–5 sessions per week, and programmes lasted from 2 to 52 weeks. Controls were generally sedentary and received only health education or minimal instruction.

Adiponectin and leptin were chosen as the primary outcomes because they capture different facets of adipose biology. Adiponectin was read as a marker of endocrine function, anti-inflammatory capacity, and insulin sensitivity (Yamauchi et al., 2002; Turer and Scherer, 2012). Leptin, in turn, indexed total fat mass and the central control of energy balance (Myers et al., 2010; Stern et al., 2016). Read together, they describe complementary parts of the metabolic and inflammatory disturbance of obesity and its cardiovascular consequences (Ouchi et al., 2011). Study-level characteristics are given in **Supplementary Table 2**.

### 3.2 Risk of bias and methodological quality

Methodological quality varied across the 34 trials (**Supplementary Figures S3**, **S4**). Random sequence generation was usually rated low risk, whereas allocation concealment and blinding of participants and personnel were the domains most often unclear or high risk — an almost unavoidable feature of exercise trials, since neither participants nor supervising staff can be blinded to training. Blinding of outcome assessment for serum adipokines was generally low risk, because assays were run in batched, blinded laboratory analyses. Incomplete outcome data and selective reporting were mostly low risk, though a few trials lost more than 20% of participants at follow-up. No trial was low risk in all seven domains, and none was high risk in most of them. The proportion of trials at each risk level by domain is shown in **Supplementary Figures S3** and **S4**.

### 3.3 Synthesis of results

#### 3.3.1 Overall effect of exercise on adiponectin and leptin

**Figure 1:**
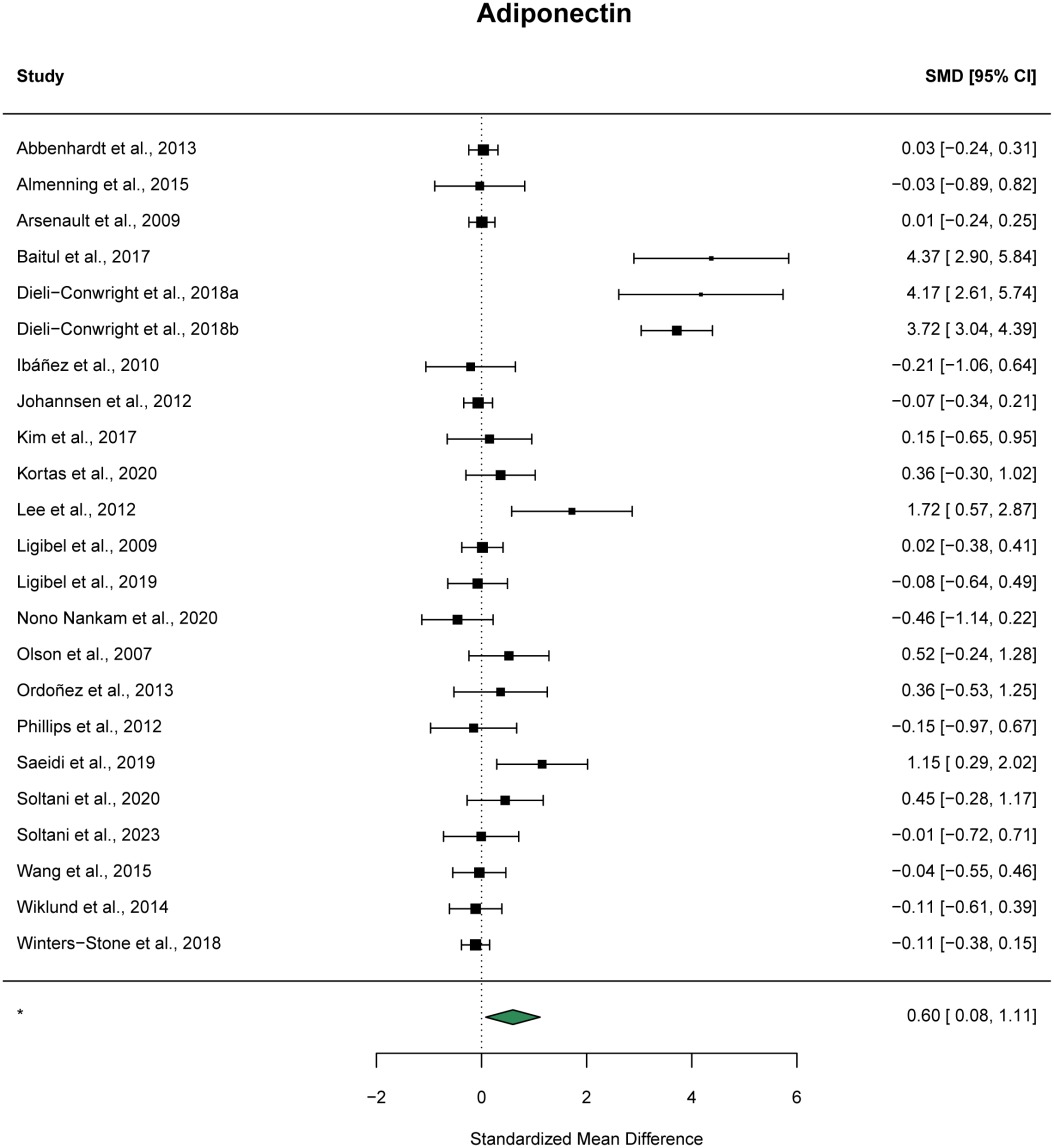
Forest plot of the change in adiponectin with exercise. Each square marks a study’s standardized mean difference (SMD), sized by its weight in the random-effects model; the horizontal bars span the 95% confidence interval (CI), and the diamond shows the pooled estimate.

**Figure 2:**
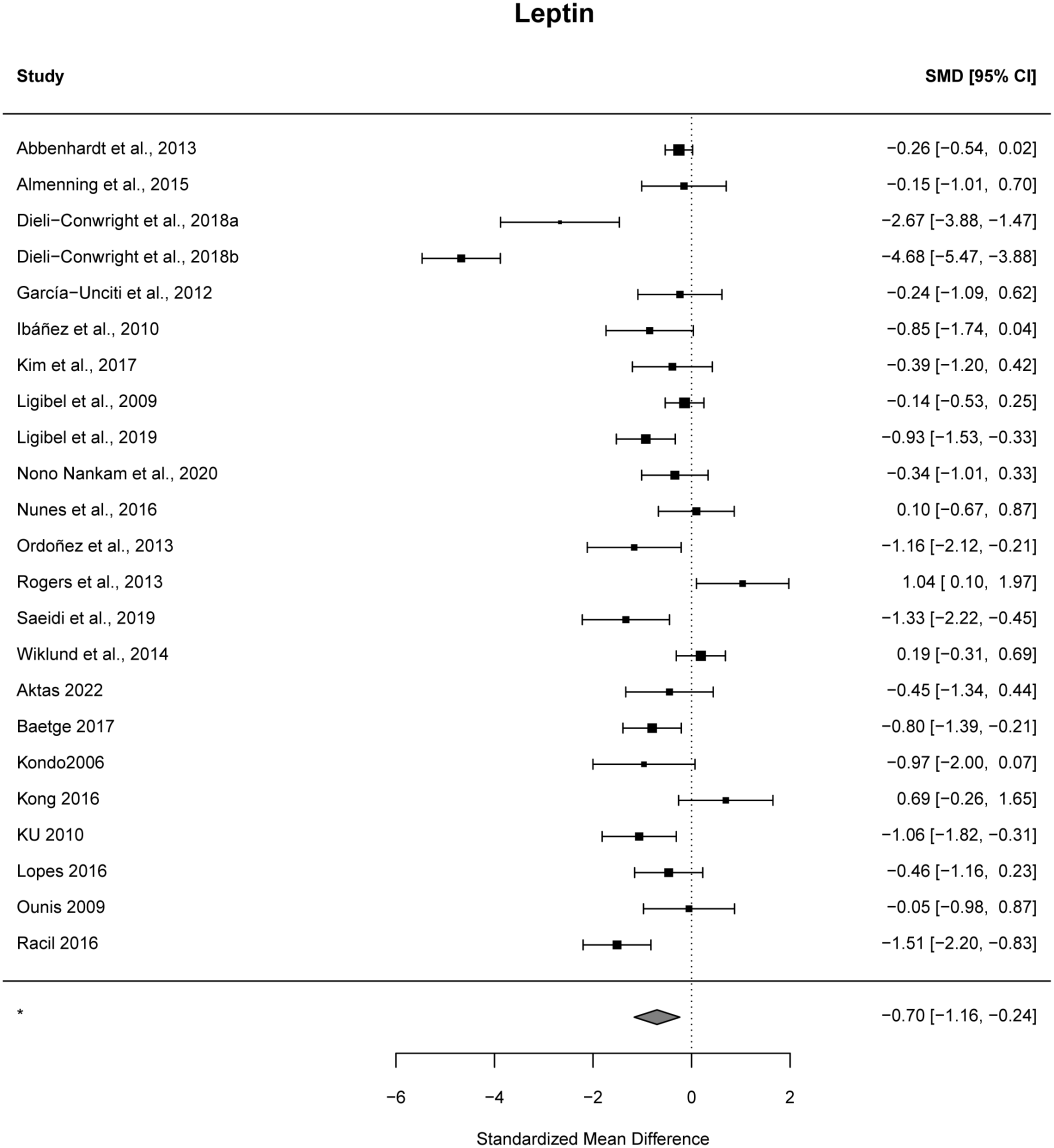
Forest plot of the change in leptin with exercise; the symbols follow the convention of **Figure 1** (SMD, standardized mean difference; CI, confidence interval).

Pooled across trials, exercise improved both adipokines but in opposite directions. As shown in **Figure 1**, adiponectin rose significantly, with a pooled SMD of 0.60 (95% CI 0.08 to 1.11, P < 0.05). Leptin fell, as shown in **Figure 2**, with a pooled SMD of -0.70 (95% CI -1.16 to -0.24, P < 0.01). Heterogeneity was high for both hormones, which prompted the meta-regression and subgroup analyses that follow.

#### 3.3.2 Meta-regression analysis (dose-response relationship)

**Figure 3:**
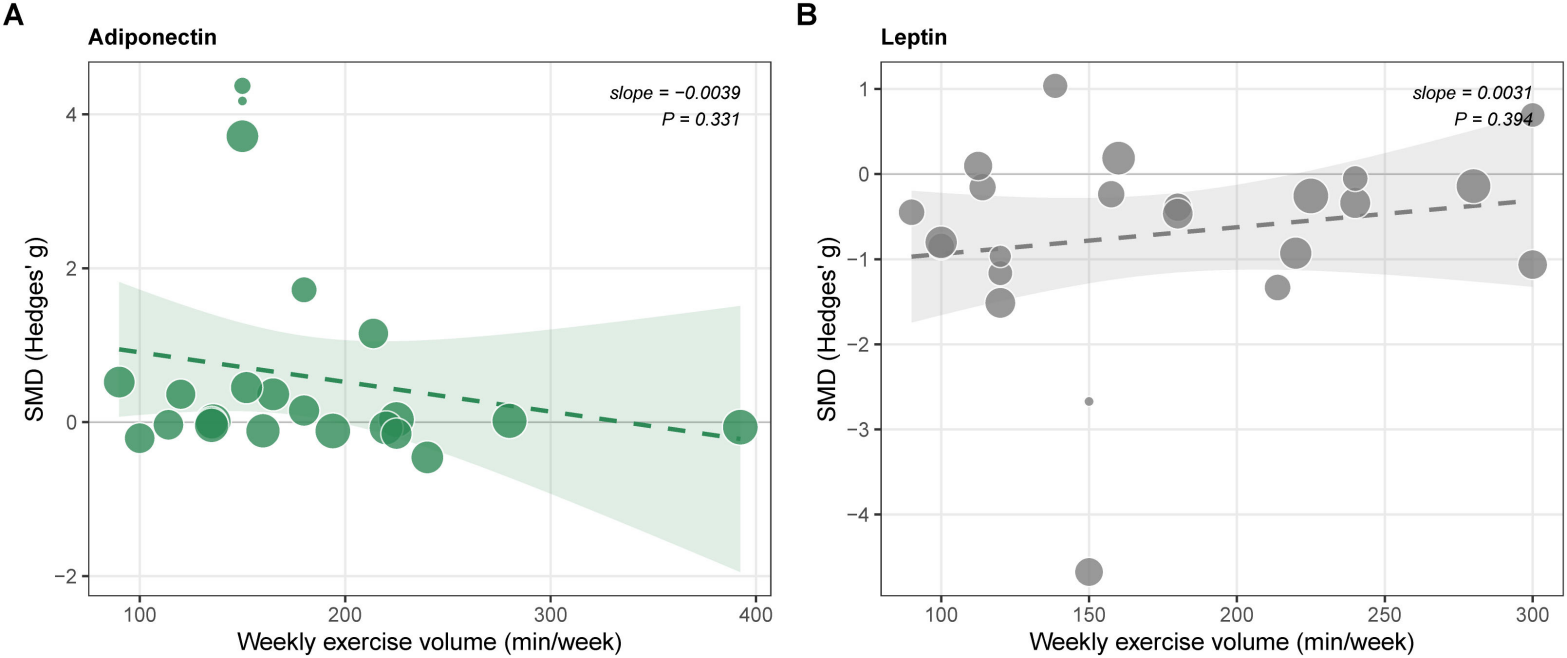
Bubble plots of the dose-response relationship between weekly exercise volume (min/week) and the effect size (SMD) for **(A)** adiponectin and **(B)** leptin. Bubble area is proportional to study weight, and the shaded band marks the 95% confidence interval. The green dashed line in **(A)** and the grey dashed line in **(B)** both denote non-significant slopes (P = 0.331 and P = 0.394, respectively).

To trace this heterogeneity and test the two predictions, weekly exercise volume (minutes per week) was entered as a continuous moderator in univariate meta-regression. As shown in **Figure 3A**, adiponectin showed no significant dose-response: weekly volume did not predict the adiponectin effect size, and the meta-regression slope was slightly negative and non-significant (slope = -0.0039, P = 0.331). The matching analysis for leptin is shown in **Figure 3B** and revealed no link between weekly volume and effect size (slope = 0.0031, P = 0.394); the effect sizes held roughly steady across the whole range of volumes. Such flatness fits a threshold response, in which the size of the leptin reduction depends on whether a sufficient stimulus is reached rather than on the total exercise accumulated.

#### 3.3.3 Subgroup analysis by exercise intensity (HIIT vs. MICT)

**Figure 4:**
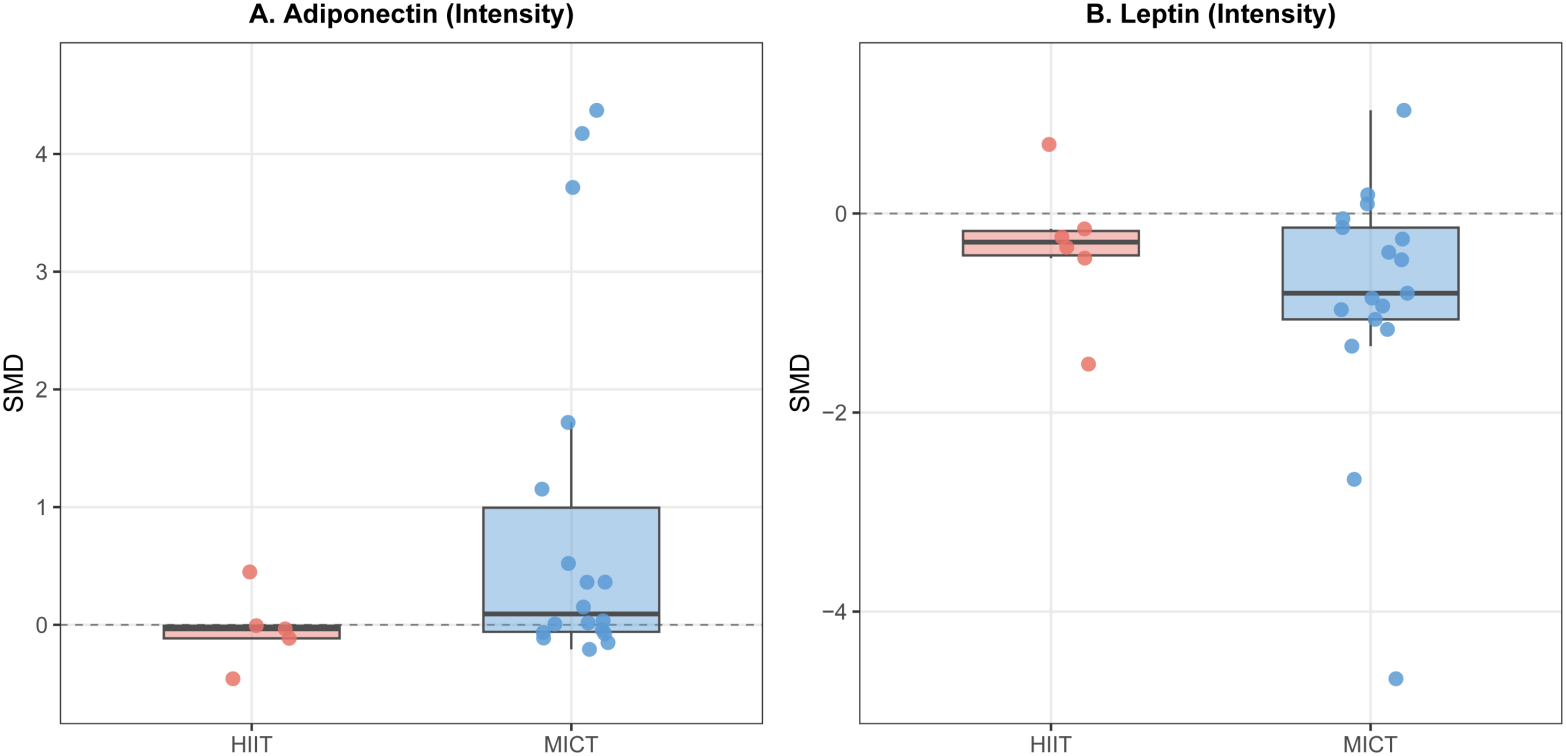
Effect sizes (SMD) by training intensity (MICT vs. HIIT) for **(A)** adiponectin and **(B)** leptin, shown as boxplots. For adiponectin the MICT effects (blue) sit higher and spread wider than the HIIT effects (red); for leptin the two intensities overlap closely.

To gauge the contribution of intensity, the trials were split into HIIT and MICT subgroups. Although weekly volume did not predict the effect size, the adiponectin subgroup analysis (**Figure 4A**) gave a larger pooled effect for MICT than for HIIT, although the formal test for subgroup differences was not significant (Q-test for subgroup heterogeneity: P > 0.05). The MICT effects were spread wider and shifted toward larger positive values than the narrower HIIT effects, consistent with the sustained duration of continuous training mattering for adiponectin. The leptin response, by contrast, looked much the same regardless of intensity (**Figure 4B**). Here the interquartile ranges overlapped substantially and the median reductions were similar for HIIT and MICT. This indifference to intensity again points to a threshold effect for leptin, with the reduction probably tied to a general negative energy balance rather than to the specific demands of high-intensity work. That link to energy balance is an interpretation rather than a tested result, since energy expenditure, intake, and net energy balance were reported too inconsistently to enter the model.

### 3.4 Publication bias

**Figure 5:**
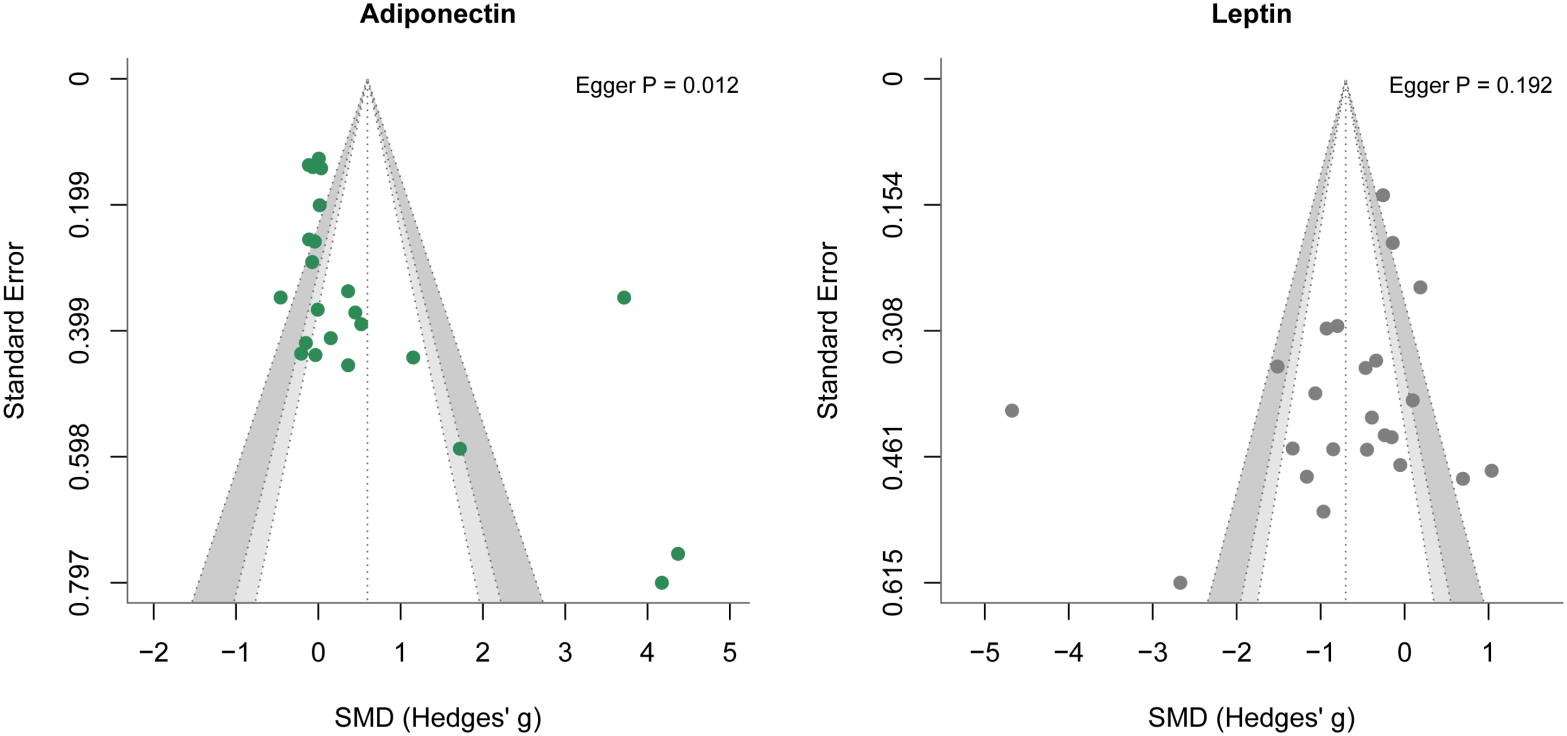
Funnel plots used to assess small-study effects for **(A)** adiponectin and **(B)** leptin; the reported P-values are from Egger’s regression test for asymmetry.

Both outcomes were checked for funnel-plot asymmetry using Egger’s regression test (Egger et al., 1997). The two hormones behaved differently. For adiponectin (**Figure 5A**), the plot was asymmetric: smaller trials reported larger positive effects, and Egger’s test was significant (P = 0.012). Asymmetry of this kind usually reflects small-study effects, which can arise from genuine between-study heterogeneity rather than from any suppression of null results (Sterne et al., 2011). By contrast, the leptin plot (**Figure 5B**) was symmetrical around the pooled estimate, and Egger’s test was not significant (P = 0.192). There is thus no sign of funnel-plot asymmetry for leptin, and the pooled reduction is unlikely to stem from selective reporting. The significant funnel asymmetry for adiponectin does not contradict the mostly low-to-unclear selective-reporting ratings in the formal risk-of-bias assessment (Section 3.2): the two assessments capture different phenomena. The Cochrane tool evaluates whether individual trialists suppressed or selectively reported outcomes, whereas funnel-plot asymmetry reflects an aggregate pattern across the literature. As Sterne et al. (2011) note, asymmetry can arise from genuine between-study heterogeneity — plausible here given the high I² for adiponectin — rather than from deliberate outcome suppression; accordingly, the adiponectin asymmetry is best interpreted as a small-study heterogeneity effect rather than as evidence of reporting bias within individual trials.

### 3.5 Sensitivity analysis

Influence diagnostics based on Cook’s distance are shown in **Supplementary Figure 5**. For adiponectin, three trials exceeded the 4/n threshold (0.193) and stood out as the most influential: Dieli-Conwright et al. (2018b), Baitul et al. (2017), and Dieli-Conwright et al. (2018a); these were also the studies with the largest positive effect estimates. For leptin, only one trial, Dieli-Conwright et al. (2018b), exceeded the threshold (0.153), with all remaining studies falling well below it. The concentration of influential studies in the adiponectin analysis is consistent with the high heterogeneity and the funnel-plot asymmetry noted above, and indicates that the pooled adiponectin estimate should be interpreted with these trials in mind.

## 4 Discussion

### 4.1 Principal findings

As far as we are aware, this is the first review to model the dose-response relationship for adiponectin and leptin separately in women with overweight or obesity. The results argue against the common assumption that adipose-derived hormones all respond to exercise in the same way. Neither hormone changed in proportion to weekly volume; instead, adiponectin increased more with moderate-intensity continuous training than with HIIT, whereas leptin fell in a threshold-like fashion that changed little with added exercise time and, on our reading, depended more on reaching a negative energy balance than on extra training minutes. These findings extend earlier reviews that reported marked heterogeneity without pinning down its source (Fedewa et al., 2018; Wang et al., 2024). Exercise helps both hormones, but the levers differ: adiponectin responded more to the type of training, favoring moderate-intensity continuous exercise over HIIT, than to the accumulated weekly minutes, while leptin responds to whether a sufficient stimulus, most likely an energy deficit, has been reached.

### 4.2 Adiponectin, exercise volume, and intensity

Contrary to our prediction, weekly volume did not predict the adiponectin effect size (slope = -0.0039, P = 0.331); instead, the larger pooled effect for MICT than HIIT points to adiponectin depending on the type and continuity of aerobic exercise rather than on the total volume accumulated. One likely mechanism is partial relief of adipose hypoxia. In obesity, fat cells enlarge faster than their blood supply can grow, creating a low-oxygen environment that stabilizes hypoxia-inducible factor 1-α (HIF-1α), a direct repressor of the adiponectin gene (ADIPOQ) (Halberg et al., 2009). Genetic variation in ADIPOQ and ADIPOR1 can also interact with dietary saturated fatty acids to modulate insulin resistance (Ferguson et al., 2010), indicating that adiponectin-related metabolic effects are shaped jointly by exercise, diet, and genotype. The benefit of sustained continuous training may therefore reflect the time needed for exercise to build new vessels. Sustained aerobic activity raises laminar shear stress, which promotes the release of endothelial nitric oxide synthase (eNOS) and vascular endothelial growth factor (VEGF) (Gleeson et al., 2011).

Activation of peroxisome proliferator-activated receptor γ (PPARγ) signaling, central to adiponectin synthesis, may likewise need prolonged metabolic stress to override the suppression exerted by pro-inflammatory cytokines such as TNF-α (Kadowaki et al., 2006). Brief HIIT sessions, however intense, may simply not provide enough total exposure to clear chronic macrophage infiltration from visceral fat or to quiet JNK-mediated inflammatory signaling. On this account, the sustained, continuous nature of moderate-intensity aerobic activity may be what lifts the hypoxic brake on adiponectin production.

### 4.3 Leptin and the threshold-type response

Leptin behaved differently. The meta-regression found no relationship with weekly volume (slope = 0.0031, P = 0.394), and reductions were comparable in MICT and HIIT trials. A flat curve of this sort fits a threshold response, with leptin acting chiefly as a gauge of energy availability and total fat mass rather than as a readout of slow tissue remodeling (Friedman, 2019). Circulating leptin falls quickly when a caloric deficit appears, often before any real loss of fat mass, through sympathetic inhibition of leptin gene expression (Rayner and Trayhurn, 2001).

Our data fit the idea that, once a sufficient energy deficit is created, whether by a short high-intensity session or a longer moderate one, further weekly minutes do little to lower leptin. This also matches reports that leptin responds weakly to exercise on its own when increased food intake offsets the energy the exercise expends (Reseland et al., 2001). For the goal of lowering leptin, and perhaps easing leptin resistance, the balance between energy spent and energy taken in may therefore matter more than the exact duration or intensity of exercise, though it is unlikely to be the only factor. This remains a hypothesis rather than a result: we could not enter energy expenditure, energy intake, weight loss, or net energy balance into the regression as moderators, because the trials reported them either not at all or too inconsistently. Settling the question will require primary studies, ideally with individual-participant data, that record these variables systematically.

### 4.4 Practical implications for exercise prescription

The contrast we observed suggests that exercise advice for women with overweight or obesity need not be uniform. When the dominant problem is low adiponectin with raised inflammatory markers, the modality of training appears to matter more than the weekly volume, and sustained moderate-intensity continuous aerobic exercise rather than brief high-intensity work is a sensible choice, since adiponectin increased more with MICT than with HIIT. When the dominant problem is high leptin with difficulty losing weight, the priority shifts to reaching and holding a negative energy balance, and time-efficient HIIT may serve just as well while being easier to keep up. This two-track approach mirrors the divergent responses seen here, although individual variation is wide and exercise should always sit alongside attention to diet and overall energy balance.

### 4.5 Strengths and limitations

The review’s main strengths are the separation of adiponectin and leptin as distinct outcomes, the modelling of weekly volume as a continuous moderator so that a dose-response slope could be estimated for each hormone, and the use of influence diagnostics to test the robustness of the pooled estimates.

Five limitations apply. First, the wide ranges of age (13.1–66.78 years), BMI (23.3–37.0 kg/m²), and menopausal status across trials meant that confounding could not be eliminated through multivariate meta-regression, as too few studies reported all covariates simultaneously; individual-participant data analyses should prioritize this gap. Second, inclusion of resistance and concurrent training alongside aerobic protocols introduces mechanistic heterogeneity that may dilute the aerobic dose-response signal. Third, adiponectin and leptin effects were not reported separately by menopausal status, so divergent responses in pre- and postmenopausal women may be masked in the pooled estimates. Fourth, PROSPERO registration post-dated the literature search, limiting its value as a prospective safeguard against outcome selection. Fifth, energy expenditure, dietary intake, and weight loss were reported too inconsistently across trials to test the energy-balance mechanism for leptin directly; that explanation therefore remains a hypothesis for future work.

## 5 Conclusion

Adiponectin and leptin both responded to exercise, but neither response scaled with the weekly volume of training in women with overweight or obesity. Adiponectin increased more with MICT than with HIIT, even though the subgroup difference fell short of significance, which suggests that the continuity of moderate-intensity training, rather than the accumulated weekly volume, is what drives it upward. Leptin, by contrast, fell in a threshold-like manner that was largely independent of both total volume and intensity; we tentatively attribute this to a negative energy balance, but energy expenditure, intake, and weight loss were reported too inconsistently to test that idea, so it should be regarded as a hypothesis for future work. These results support tailoring exercise to the goal. To raise adiponectin and reduce systemic inflammation, moderate-intensity continuous aerobic training is the more reasonable choice; to lower leptin and aid weight regulation, MICT and shorter HIIT appear comparable, provided that overall energy balance is also addressed.

## Data Availability

The original contributions presented in the study are included in the article/**Supplementary Material**. Further inquiries can be directed to the corresponding authors.
