# Supplementary material for: Divergent responses of adiponectin and leptin to exercise in women with overweight or obesity: a systematic review and meta-regression
Source: Front Physiol. 2026 Jul 15;17:1810408. doi: 10.3389/fphys.2026.1810408 (PMC13414222; doi:10.3389/fphys.2026.1810408)
Supplement: Supplementary file 1 [file SupplementaryFile1.docx]

**Supplemental material**

| **Content** | **Pages** |
| --- | --- |
| **Figure S1.** Search strategy used in the Cochrane Library. | 2 |
| **Table S1.** Eligibility criteria based on the PICOS framework | 2 |
| **Figure S2.** Flow chart of literature screening | 3 |
| **Figure S3.** Summary of the risk of bias assessment | 3 |
| **Figure S4.** Risk of bias assessment results | 4 |
| **Figure S5.** Influence diagnostics (Cook’s distance) | 5 |
| **Table S2.** Characteristics of included studies | 5 - 6 |
| **Table S3.** PRISMA 2020 Checklist | 7 - 9 |
| **References** | 10 - 12 |

**Figure S1. Search strategy used in the Cochrane Library**


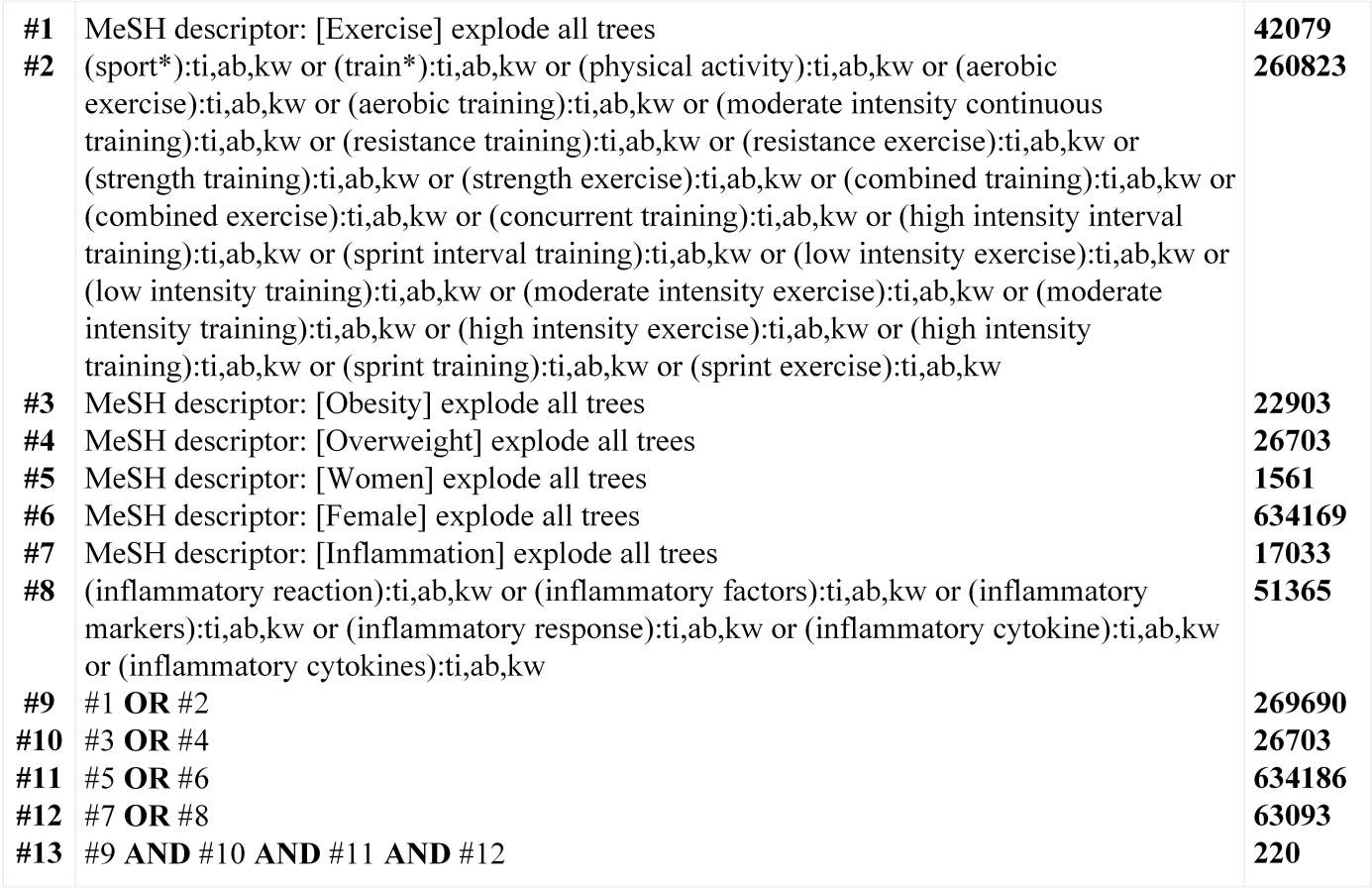


**Table S1.** Eligibility criteria based on the PICOS framework

| Component | Inclusion Criteria | Exclusion Criteria |
| --- | --- | --- |
| Participants | Females (aged ≥ 13 years); Overweight (BMI ≥ 23 kg/m²) or Obese (BMI ≥ 30 kg/m²) | Professional athletes; Pregnant women∙ Participants with severe chronic diseases (e.g., cancer, heart failure) that contraindicate exercise |
| Interventions | Structured exercise training programs (e.g., aerobic, resistance, HIIT, or combined training); Duration of at least 2 weeks | Acute exercise interventions (single session); Combined interventions (e.g., exercise + diet or medication) where the independent effect of exercise cannot be isolated |
| Comparators | Non-exercising control group (e.g., sedentary, wait-list, or usual care) | (No specific exclusion criteria stated separate from intervention confounding) |
| Outcomes | Pre- and post-intervention data on circulating levels of adiponectin and/or leptin | Insufficient data for effect size calculation (e.g., missing mean or standard deviation) |
| Study Design | Randomized Controlled Trials (RCTs); Published in English | Non-randomized trials, observational studies, reviews, or animal studies |

**Figure S2.** Flow chart of literature screening


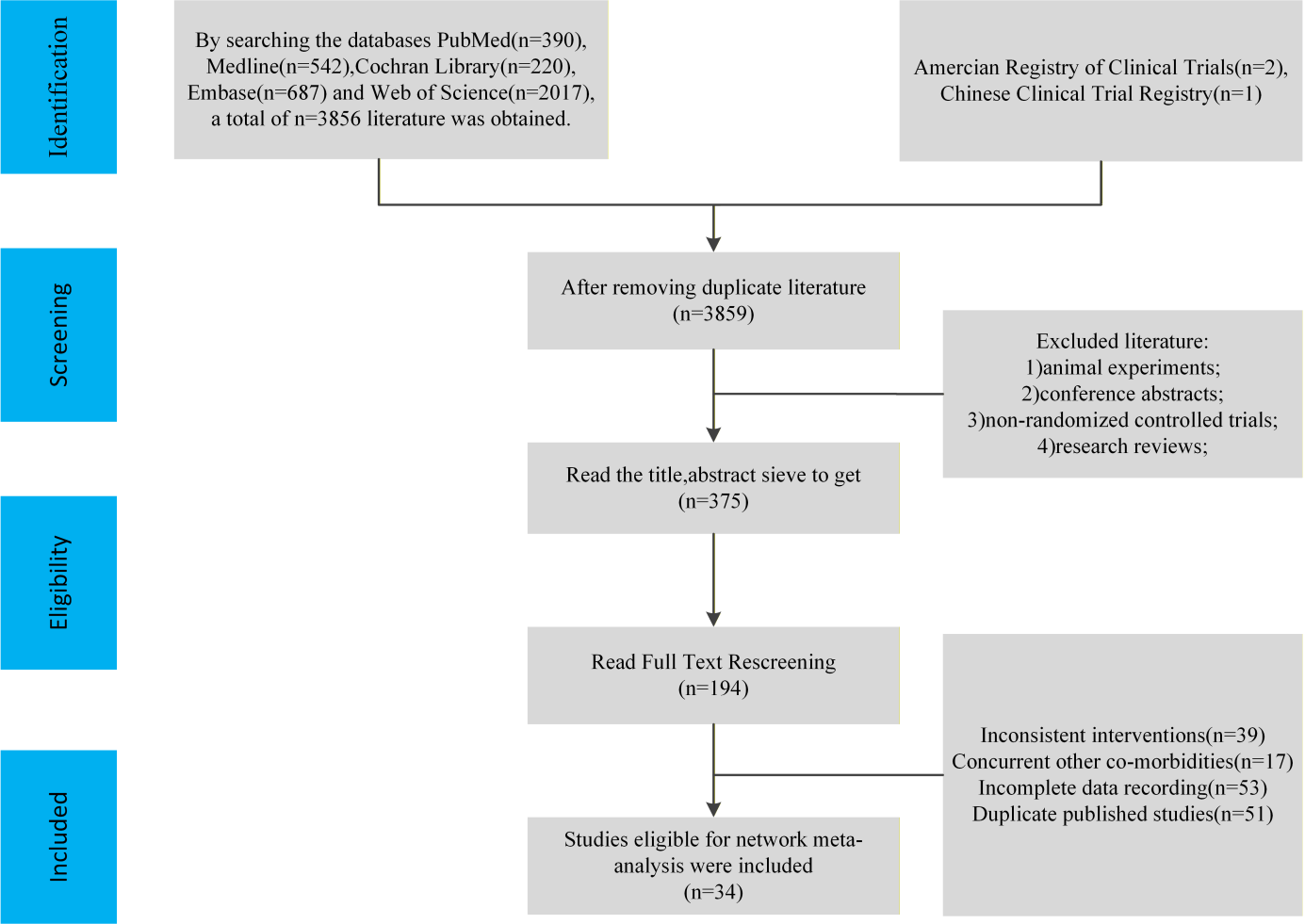


**Figure S3.** Summary of the risk of bias assessment


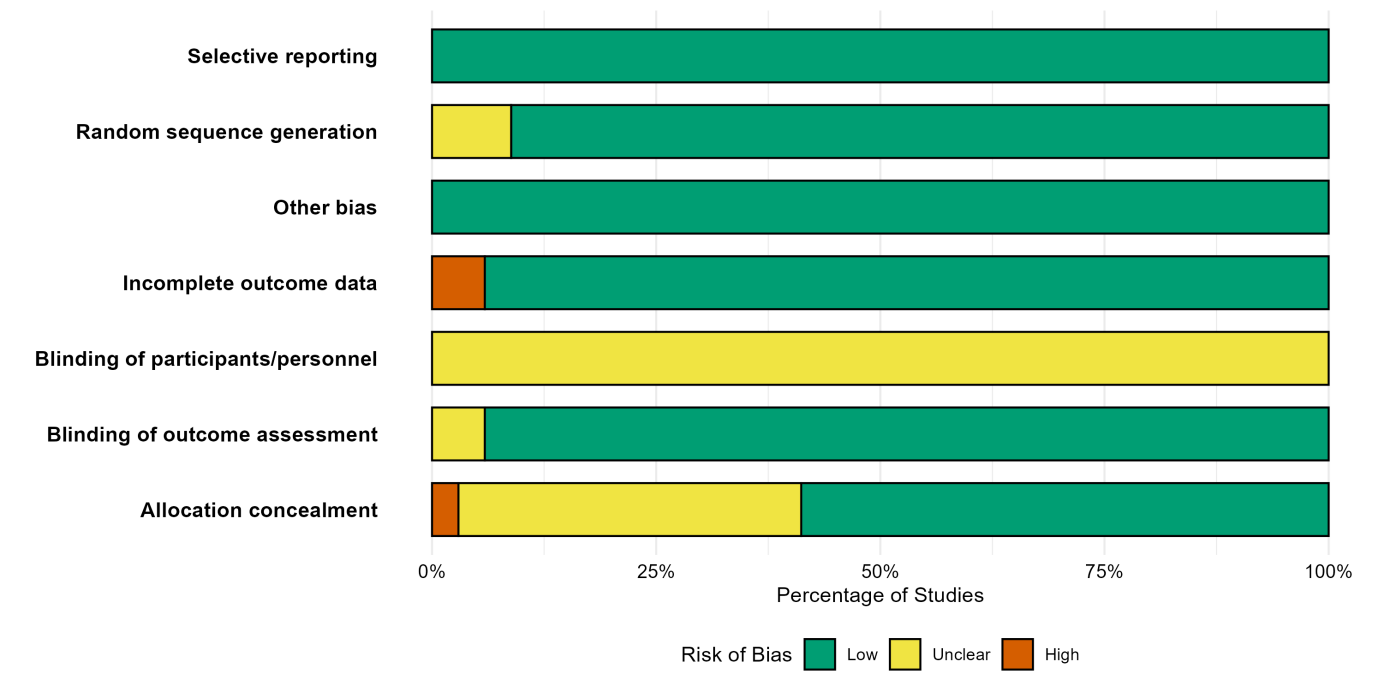


**Figure S4.** Risk of bias assessment results


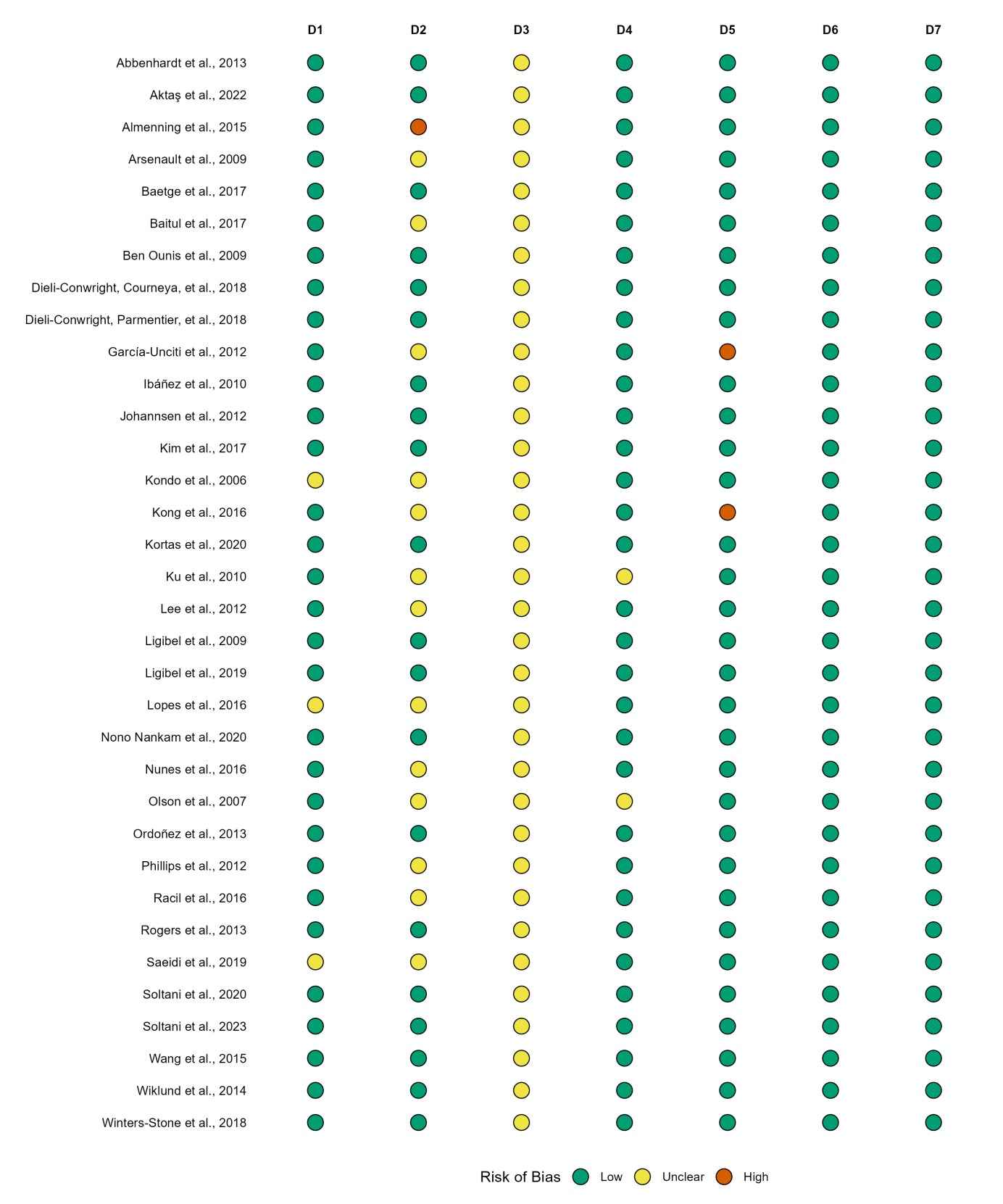


**Figure S5.** Influence diagnostics (Cook’s distance) for each trial in the (A) adiponectin and (B) leptin meta-analyses. The dashed vertical line marks the 4/n threshold (0.193 for adiponectin and 0.153 for leptin); studies exceeding it, shown in red, were flagged as potentially influential.


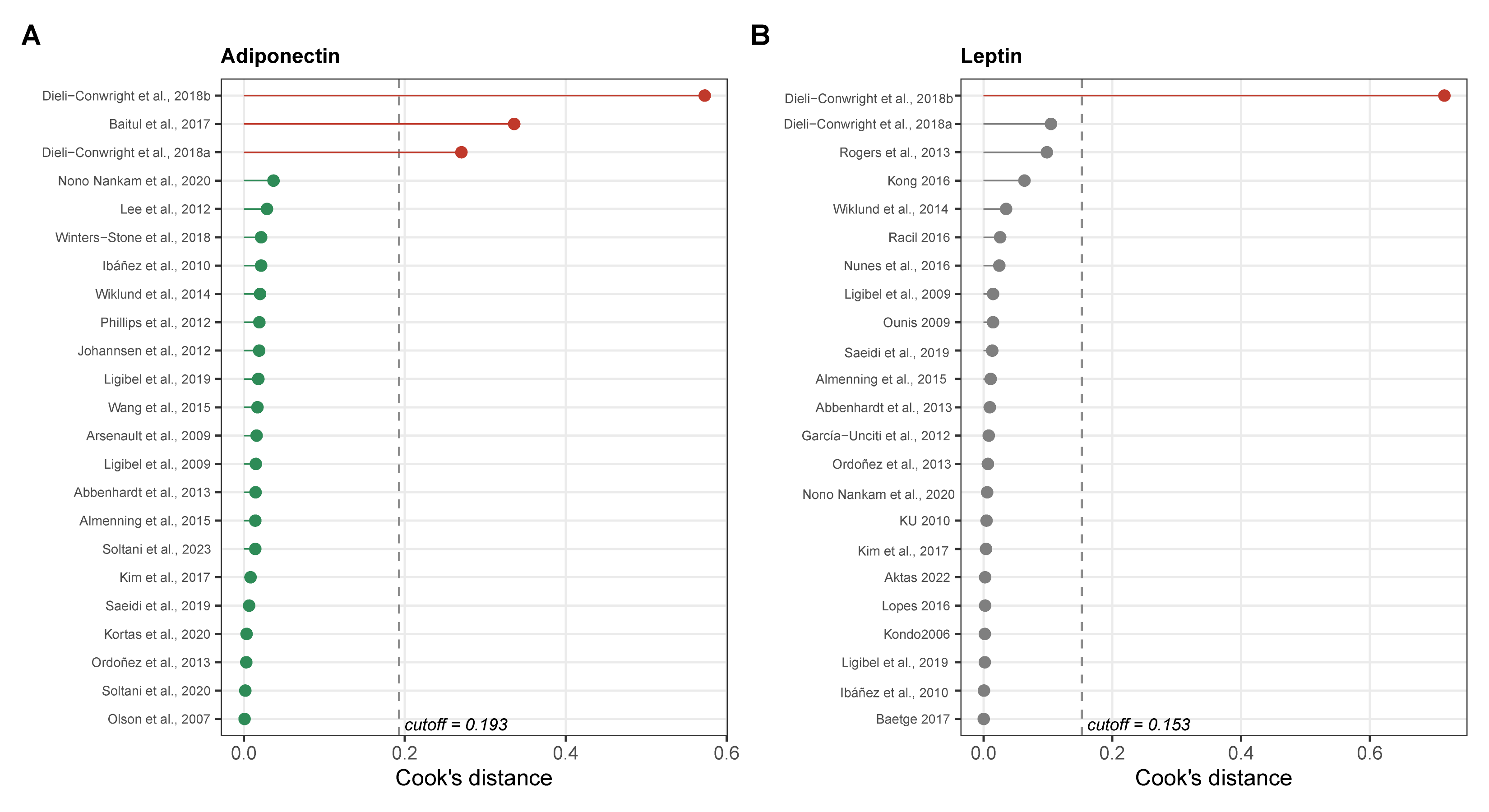


**Table S2.** Characteristics of included studies

| **Study** | **Country** | **Participant Characteristics (Exp/Ctrl)** | | | **Intervention Protocol Exp Group** | | | | | **Outcomes** |
| --- | --- | --- | --- | --- | --- | --- | --- | --- | --- | --- |
|  |  | **N** | **Age (years)** | **BMI kg/m²** | Weeks | Freq | Session_Min | Mode | Intensity |  |
| Abbenhardt et al., 2013 | USA | 117 / 87 | 58.1 ± 5.0 / 57.4 ± 4.4 | > 25 / > 25 | 52 | 5 | 45 | AT | Moderate | Adiponectin, Leptin |
| Aktaş et al., 2022 | Turkey | 10 / 10 | 25.1 ± 4.6 / 24.6 ± 6.7 | 28.7 ± 6.9 / 28.6 ± 4.9 | 12 | 3 | 30 | HIIT | HIIT | Leptin |
| Almenning et al., 2015 | Norway | 11 / 10 | 27.2 ± 5.5 / 27.2 ± 5.5 | 27.4 ± 6.9 / 26.5 ± 5.0 | 10 | 3 | 38 | HIIT | HIIT | Adiponectin, Leptin |
| Arsenault et al., 2009 | USA | 267 / 82 | 57.3 ± 6.6 / 57.2 ± 6.1 | 32.0 ± 5.7 / 31.9 ± 3.8 | 26 | 3.5 | 38.8 | AT | Moderate | Adiponectin |
| Baetge et al., 2017 | USA | 29 / 20 | 47 ± 10 / 51 ± 11 | 35 ± 6 / 37 ± 5 | 12 | 2 | 50 | CT | Moderate | Leptin |
| Baitul et al., 2017 | Indonesia | 12 / 12 | 46.74 ± 1.30 / 46.79 ± 1.35 | 31.32 ± 0.97 / 32.48 ± 1.56 | 8 | 3 | 50 | AT | Moderate | Adiponectin |
| Ben Ounis et al., 2009 | Tunisie | 9 / 9 | 13.1 ± 0.9 / 13.2 ± 0.3 | 30.4 ± 1.8 / 30.5 ± 2.2 | 8 | 4 | 60 | AT | Moderate | Leptin |
| Dieli-Conwright et al., 2018a | USA | 10 / 10 | 53.0 ± 10.0 / 55.0 ± 4.5 | 33.5 ± 5.7 / 33.3 ± 8.7 | 16 | 3 | 50 | CT | Moderate | Adiponectin, Leptin |
| Dieli-Conwright et al., 2018b | USA | 46 / 45 | 52.8 ± 10.6 / 53.6 ± 10.1 | 33.5 ± 5.7 / 33.7 ± 5.2 | 16 | 3 | 50 | CT | Moderate | Adiponectin, Leptin |
| García-Unciti et al., 2012 | Spain | 13 / 9 | 48.6 ± 6.4 / 50.2 ± 6.8 | 35 ± 3.1 / 35 ± 3.6 | 16 | 2 | 78.75 | RT | HIIT | Leptin |
| Ibáñez et al., 2010 | Spain | 13 / 9 | 48.6 ± 6.4 / 50.2 ± 6.8 | 35 ± 3.1 / 35 ± 3.6 | 16 | 2 | 50 | RT | Moderate | Adiponectin, Leptin |
| Johannsen et al., 2012 | USA | 103 / 102 | 56.6 ± 6.5 / 57.1 ± 5.7 | 31.2 ± 3.5 / 31.7 ± 3.8 | 9 | 4 | 98.08 | AT | Moderate | Adiponectin |
| Kim et al., 2017 | Korea | 11 / 13 | 56.0 ± 6.5 / 49.3 ± 4.8 | 23.9 ± 2.7 / 25.0 ± 4.7 | 12 | 3 | 60 | CT | Moderate | Adiponectin, Leptin |
| Kondo et al., 2006 | Japan | 8 / 8 | 18.0 ± 1.0 / 18.0 ± 1.5 | 29.5 ± 2.7 / 21.9 ± 3.2 | 28 | 2 | 60 | AT | Moderate | Leptin |
| Kong et al., 2016 | China | 10 / 8 | 19.8 ± 0.8 / 19.9 ± 2.1 | 25.5 ± 2.1 / 26.2 ± 2.4 | 5 | 5 | 60 | HIIT | HIIT | Leptin |
| Kortas et al., 2020 | Poland | 18 / 18 | 66.78 ± 4.76 / 66.12 ± 4.83 | 25.87 ± 3.45 / 25.96 ± 5.12 | 12 | 3 | 55 | AT | Moderate | Adiponectin |
| Ku et al., 2010 | Korea | 15 / 16 | 55.7 ± 7.0 / 57.8 ± 8.1 | 27.1 ± 2.4 / 27.4 ± 2.8 | 12 | 5 | 60 | RT | Moderate | Leptin |
| Lee et al., 2012 | Korea | 8 / 8 | 54.75 ± 2.76 / 54.25 ± 2.91 | 25.13 ± 1.63 / 25.19 ± 1.71 | 16 | 3 | 60 | AT | Moderate | Adiponectin |
| Ligibel et al., 2009 | USA | 51 / 49 | 52 ± 9 / 53 ± 9 | 30.3 ± 5.9 / 31.4 ± 6.8 | 16 | 2 | 140 | CT | Moderate | Adiponectin, Leptin |
| Ligibel et al., 2019 | USA | 26 / 22 | 52.3 ± 9.6 / 53.1 ± 7.9 | 30.7 ± 6.1 / 29.1 ± 7.4 | 15 | 3 | 73.3 | CT | Moderate | Adiponectin, Leptin |
| Lopes et al., 2016 | Brazil | 17 / 15 | 14.6 ± 1.15 / 14.4 ± 1.16 | 28.8 ± 3.62 / 29.4 ± 3.09 | 12 | 3 | 60 | CT | Moderate | Leptin |
| Nono Nankam et al., 2020 | South Africa | 20 / 15 | 20–30 / 20–30 | 34.1 ± 0.6 / 33.4 ± 0.7 | 12 | 4 | 60 | CT | HIIT | Adiponectin, Leptin |
| Nunes et al., 2016 | Brazil | 11 / 11 | 54.7–65.5 / 54.0–64.5 | 23.3–33.7 / 25.2–33.6 | 16 | 3 | 37.5 | RT | Moderate | Leptin |
| Olson et al., 2007 | USA | 16 / 12 | 39 ± 5 / 38 ± 6 | 26.9 ± 3.0 / 27.0 ± 3.0 | 52 | 2 | 45.03 | RT | Moderate | Adiponectin |
| Ordoñez et al., 2013 | Spain | 11 / 9 | 24.7 ± 3.6 / 25.1 ± 3.9 | 30.2 ± 0.9 / 30.7 ± 0.8 | 10 | 3 | 40 | AT | Moderate | Adiponectin, Leptin |
| Phillips et al., 2012 | USA | 11 / 12 | 64.8 ± 2.4 / 66.4 ± 2.8 | 32.2 ± 3.3 / 33.7 ± 4.4 | 12 | 3 | 75 | RT | Moderate | Adiponectin |
| Racil et al., 2016 | Tunisia | 23 / 19 | 16.6 ± 0.9 / 16.9 ± 1.0 | NR | 12 | 3 | 40 | HIIT | HIIT | Leptin |
| Rogers et al., 2013 | USA | 11 / 9 | 58.0 ± 6.1 / 53.7 ± 13.9 | 33.9 ± 7.4 / 30.3 ± 7.11 | 13 | 2 | 69.28 | CT | Moderate | Leptin |
| Saeidi et al., 2019 | Iran | 12 / 12 | 58 ± 5 / 56 ± 5 | 27.2 ± 1.8 / 28.2 ± 1.9 | 8 | 3 | 71.25 | RT | Moderate | Adiponectin, Leptin |
| Soltani et al., 2020 | Iran | 15 / 15 | 18–25 / 18–25 | 31.1 ± 4.2 / 30.7 ± 4.3 | 10 | 4 | 38 | HIIT | HIIT | Adiponectin |
| Soltani et al., 2023 | Iran | 15 / 15 | 21 ± 1.4 / 20.7 ± 1.5 | 30.6 ± 3.7 / 31.1 ± 4.8 | 2 | 3 | 45 | HIIT | HIIT | Adiponectin |
| Wang et al., 2015 | USA | 48 / 22 | 58.4 ± 5.2 / 58.5 ± 6.1 | 32.9 ± 3.7 / 28.7 ± 3.5 | 20 | 3 | 45 | AT | Moderate | Adiponectin |
| Wiklund et al., 2014 | Finland | 31 / 31 | 41.9 ± 7.3 / 42.2 ± 7.5 | 28.4 ± 2.1 / 31.3 ± 3.1 | 6 | 2 | 80 | AT | Moderate | Adiponectin, Leptin |
| Winters-Stone et al., 2018 | USA | 109 / 106 | 59.8 ± 11.4 / 59.3 ± 11.6 | 27.9 ± 5.5 / 28.5 ± 5.3 | 52 | 2 | 97 | RT | HIIT | Adiponectin |

**Note:** Data are expressed as mean ± standard deviation (SD) or range (min–max) unless otherwise indicated. Values separated by a forward slash (e.g., Exp/Ctrl) denote the Experimental group and Control group, respectively. Abbreviations: AT, aerobic training; BMI, body mass index; CT, concurrent (combined) training; Ctrl, control group; d, day(s); Exp, experimental group; HIIT, high-intensity interval training; min, minute(s); NR, not reported; RT, resistance training; wk, week(s).

**Table S3.** PRISMA 2020 Checklist

| **Section/Topic** | **Item #** | **Checklist Item** | **Reported on Page #** |
| --- | --- | --- | --- |
| **TITLE** |  |  |  |
| Title | 1 | Identify the report as a systematic review. | 1 |
| **ABSTRACT** |  |  |  |
| Abstract | 2 | See the PRISMA 2020 for Abstracts checklist. | 2 - 3 |
| **INTRODUCTION** |  |  |  |
| Rationale | 3 | Describe the rationale for the review in the context of existing knowledge. | 3 - 7 |
| Objectives | 4 | Provide an explicit statement of the objective(s) or question(s) the review addresses. | 7 |
| **METHODS** |  |  |  |
| Eligibility criteria | 5 | Specify the inclusion and exclusion criteria for the review and how studies were grouped for the syntheses. | 9 & Table S1 |
| Information sources | 6 | Specify all databases, registers, websites, organisations, reference lists and other sources searched or consulted to identify studies. Specify the date when each source was last searched or consulted. | 8 |
| Search strategy | 7 | Present the full search strategies for all databases, registers and websites, including any filters and limits used. | 8 & Figure S1 |
| Selection process | 8 | Specify the methods used to decide whether a study met the inclusion criteria of the review, including how many reviewers screened each record and each report retrieved, whether they worked independently, and if applicable, details of automation tools used in the process. | 9 & Figure S2 |
| Data collection process | 9 | Specify the methods used to collect data from reports, including how many reviewers collected data from each report, whether they worked independently, any processes for obtaining or confirming data from study investigators, and if applicable, details of automation tools used in the process. | 9 |
| Data items | 10a | List and define all outcomes for which data were sought. Specify whether all results that were compatible with each outcome domain in each study were sought (e.g. for all measures, time points, analyses), and if not, the methods used to decide which results to collect. | 9 |
|  | 10b | List and define all other variables for which data were sought (e.g. participant and intervention characteristics, funding sources). Describe any assumptions made about any missing or unclear information. | 9 |
| Study risk of bias assessment | 11 | Specify the methods used to assess risk of bias in the included studies, including details of the tool(s) used, how many reviewers assessed each study and whether they worked independently, and if applicable, details of automation tools used in the process. | 9 - 10 |
| Effect measures | 12 | Specify for each outcome the effect measure(s) (e.g. risk ratio, mean difference) used in the synthesis or presentation of results. | 11 - 12 |
| Synthesis methods | 13a | Describe the processes used to decide which studies were eligible for each synthesis (e.g. tabulating the study intervention characteristics and comparing against the planned groups for each synthesis (item #5)). | 11 - 12 |
|  | 13b | Describe any methods required to prepare the data for presentation or synthesis, such as handling of missing summary statistics, or data conversions. | 11 - 12 |
|  | 13c | Describe any methods used to tabulate or visually display results of individual studies and syntheses. | 11 - 12 |
|  | 13d | Describe any methods used to synthesize results and provide a rationale for the choice(s). If meta-analysis was performed, describe the model(s), method(s) to identify the presence and extent of statistical heterogeneity, and software package(s) used. | 11 - 12 |
|  | 13e | Describe any methods used to explore possible causes of heterogeneity among study results (e.g. subgroup analysis, meta-regression). | 11 - 12 |
|  | 13f | Describe any sensitivity analyses conducted to assess robustness of the synthesized results. | 11 - 12 |
| Reporting bias assessment | 14 | Describe any methods used to assess risk of bias due to missing results in a synthesis (arising from reporting biases). | 11 - 12 |
| Certainty assessment | 15 | Describe any methods used to assess certainty (or confidence) in the body of evidence for an outcome. | Not assessed |
| **RESULTS** |  |  |  |
| Study selection | 16a | Describe the results of the search and selection process, from the number of records identified in the search to the number of studies included in the review, ideally using a flow diagram. | 11 - 12 & Figure S2 |
|  | 16b | Cite studies that might appear to meet the inclusion criteria, but which were excluded, and explain why they were excluded. | Figure S2 |
| Study characteristics | 17 | Cite each included study and present its characteristics. | 11 - 12 & Table S2 |
| Risk of bias in studies | 18 | Present assessments of risk of bias for each included study. | 12 & Figures S3 - S4 |
| Results of individual studies | 19 | For all outcomes, present, for each study: (a) summary statistics for each group (where appropriate) and (b) an effect estimate and its precision (e.g. confidence/credible interval), ideally using structured tables or plots. | Figures 1 & 2 |
| Results of syntheses | 20a | For each synthesis, briefly summarise the characteristics and risk of bias among contributing studies. | 13 - 15 & Figures 3 - 4 & Figure S5 |
|  | 20b | Present results of all statistical syntheses conducted. If meta-analysis was done, present for each the summary estimate and its precision (e.g. confidence/credible interval) and measures of statistical heterogeneity. If comparing groups, describe the direction of the effect. | 13 - 15 & Figures 3 - 4 & Figure S5 |
|  | 20c | Present results of all investigations of possible causes of heterogeneity among study results. | 13 - 15 & Figures 3 - 4 & Figure S5 |
|  | 20d | Present results of all sensitivity analyses conducted to assess the robustness of the synthesized results. | 13 - 15 & Figures 3 - 4 & Figure S5 |
| Reporting biases | 21 | Present assessments of risk of bias due to missing results (arising from reporting biases) for each synthesis assessed. | 13 - 16 & Figure 5 |
| Certainty of evidence | 22 | Present assessments of certainty (or confidence) in the body of evidence for each outcome assessed. | Not assessed |
| **DISCUSSION** |  |  |  |
| Discussion | 23a | Provide a general interpretation of the results in the context of other evidence. | 16 - 19 |
|  | 23b | Discuss any limitations of the evidence included in the review. | 19 - 20 |
|  | 23c | Discuss any limitations of the review processes used. | 19 - 20 |
|  | 23d | Discuss implications of the results for practice, policy, and future research. | 19 - 20 |
| **OTHER INFORMATION** | |  |  |
| Registration and protocol | 24a | Provide registration information for the review, including the register name and registration number, or state that the review was not registered. | 2 & 7 |
|  | 24b | Indicate where the review protocol can be accessed, or state that a protocol was not prepared. | 7 |
|  | 24c | Describe and explain any amendments to information provided at registration or in the protocol. | 7 |
| Support | 25 | Describe sources of financial or non-financial support for the review, and the role of the funders or sponsors in the review. | 21 |
| Competing interests | 26 | Declare any competing interests of review authors. | 21 |
| Availability of data, code and other materials | 27 | Report which of the following are publicly available and where they can be found: template data collection forms; data extracted from included studies; data used for all analyses; analytic code; any other materials used in the review. | 21 |

References

Abbenhardt, C., McTiernan, A., Alfano, C.M., Wener, M.H., Campbell, K.L., Duggan, C., Foster-Schubert, K.E., Kong, A., Toriola, A.T., Potter, J.D., Mason, C., Xiao, L., Blackburn, G.L., Bain, C., and Ulrich, C.M. (2013). Effects of individual and combined dietary weight loss and exercise interventions in postmenopausal women on adiponectin and leptin levels. *J Intern Med* 274**,** 163-175.

Aktaş, H., Uzun, Y.E., Kutlu, O., Pençe, H.H., Özçelik, F., Çil, E., Irak, L., Altun, Ö., Özcan, M., Özsoy, N., Aydın Yoldemir, Ş., Kalyon, S., Arman, Y., and Tükek, T. (2022). The effects of high intensity-interval training on vaspin, adiponectin and leptin levels in women with polycystic ovary syndrome. *Arch Physiol Biochem* 128**,** 37-42.

Almenning, I., Rieber-Mohn, A., Lundgren, K.M., Shetelig Løvvik, T., Garnæs, K.K., and Moholdt, T. (2015). Effects of High Intensity Interval Training and Strength Training on Metabolic, Cardiovascular and Hormonal Outcomes in Women with Polycystic Ovary Syndrome: A Pilot Study. *PLoS One* 10**,** e0138793.

Arsenault, B.J., Côté, M., Cartier, A., Lemieux, I., Després, J.P., Ross, R., Earnest, C.P., Blair, S.N., and Church, T.S. (2009). Effect of exercise training on cardiometabolic risk markers among sedentary, but metabolically healthy overweight or obese post-menopausal women with elevated blood pressure. *Atherosclerosis* 207**,** 530-533.

Baetge, C., Earnest, C.P., Lockard, B., Coletta, A.M., Galvan, E., Rasmussen, C., Levers, K., Simbo, S.Y., Jung, Y.P., Koozehchian, M., Oliver, J., Dalton, R., Sanchez, B., Byrd, M.J., Khanna, D., Jagim, A., Kresta, J., Greenwood, M., and Kreider, R.B. (2017). Efficacy of a randomized trial examining commercial weight loss programs and exercise on metabolic syndrome in overweight and obese women. *Appl Physiol Nutr Metab* 42**,** 216-227.

Baitul, M.S., Susanto, H., Kushartanti, W., Soegiyanto, and Rahayu, S. (2017). Beneficial Health Effect of Aquarobics (Role of Adiponectin on Women with Obesity). *IOP Conference Series: Materials Science and Engineering* 180**,** 012170 (012177pp).

Ben Ounis, O., Elloumi, M., Lac, G., Makni, E., Van Praagh, E., Zouhal, H., Tabka, Z., and Amri, M. (2009). Two-month effects of individualized exercise training with or without caloric restriction on plasma adipocytokine levels in obese female adolescents. *Ann Endocrinol (Paris)* 70**,** 235-241.

Dieli-Conwright, C.M., Courneya, K.S., Demark-Wahnefried, W., Sami, N., Lee, K., Buchanan, T.A., Spicer, D.V., Tripathy, D., Bernstein, L., and Mortimer, J.E. (2018a). Effects of Aerobic and Resistance Exercise on Metabolic Syndrome, Sarcopenic Obesity, and Circulating Biomarkers in Overweight or Obese Survivors of Breast Cancer: A Randomized Controlled Trial. *J Clin Oncol* 36**,** 875-883.

Dieli-Conwright, C.M., Parmentier, J.H., Sami, N., Lee, K., Spicer, D., Mack, W.J., Sattler, F., and Mittelman, S.D. (2018b). Adipose tissue inflammation in breast cancer survivors: effects of a 16-week combined aerobic and resistance exercise training intervention. *Breast Cancer Res Treat* 168**,** 147-157.

García-Unciti, M., Izquierdo, M., Idoate, F., Gorostiaga, E., Grijalba, A., Ortega-Delgado, F., Martínez-Labari, C., Moreno-Navarrete, J.M., Forga, L., Fernández-Real, J.M., and Ibáñez, J. (2012). Weight-loss diet alone or combined with progressive resistance training induces changes in association between the cardiometabolic risk profile and abdominal fat depots. *Ann Nutr Metab* 61**,** 296-304.

Ibáñez, J., Izquierdo, M., Martínez-Labari, C., Ortega, F., Grijalba, A., Forga, L., Idoate, F., García-Unciti, M., Fernández-Real, J.M., and Gorostiaga, E.M. (2010). Resistance training improves cardiovascular risk factors in obese women despite a significative decrease in serum adiponectin levels. *Obesity (Silver Spring)* 18**,** 535-541.

Johannsen, N.M., Swift, D.L., Johnson, W.D., Dixit, V.D., Earnest, C.P., Blair, S.N., and Church, T.S. (2012). Effect of different doses of aerobic exercise on total white blood cell (WBC) and WBC subfraction number in postmenopausal women: results from DREW. *PLoS One* 7**,** e31319.

Kim, T.H., Chang, J.S., Park, K.S., Park, J., Kim, N., Lee, J.I., and Kong, I.D. (2017). Effects of exercise training on circulating levels of Dickkpof-1 and secreted frizzled-related protein-1 in breast cancer survivors: A pilot single-blind randomized controlled trial. *PLoS One* 12**,** e0171771.

Kondo, T., Kobayashi, I., and Murakami, M. (2006). Effect of exercise on circulating adipokine levels in obese young women. *Endocr J* 53**,** 189-195.

Kong, Z., Sun, S., Liu, M., and Shi, Q. (2016). Short-Term High-Intensity Interval Training on Body Composition and Blood Glucose in Overweight and Obese Young Women. *J Diabetes Res* 2016**,** 4073618.

Kortas, J., Ziemann, E., Juszczak, D., Micielska, K., Kozłowska, M., Prusik, K., Prusik, K., and Antosiewicz, J. (2020). Iron Status in Elderly Women Impacts Myostatin, Adiponectin and Osteocalcin Levels Induced by Nordic Walking Training. *Nutrients* 12.

Ku, Y.H., Han, K.A., Ahn, H., Kwon, H., Koo, B.K., Kim, H.C., and Min, K.W. (2010). Resistance exercise did not alter intramuscular adipose tissue but reduced retinol-binding protein-4 concentration in individuals with type 2 diabetes mellitus. *J Int Med Res* 38**,** 782-791.

Lee, J.A., Kim, J.W., and Kim, D.Y. (2012). Effects of yoga exercise on serum adiponectin and metabolic syndrome factors in obese postmenopausal women. *Menopause* 19**,** 296-301.

Ligibel, J.A., Dillon, D., Giobbie-Hurder, A., McTiernan, A., Frank, E., Cornwell, M., Pun, M., Campbell, N., Dowling, R.J.O., Chang, M.C., Tolaney, S., Chagpar, A.B., Yung, R.L., Freedman, R.A., Dominici, L.S., Golshan, M., Rhei, E., Taneja, K., Huang, Y., Brown, M., Winer, E.P., Jeselsohn, R., and Irwin, M.L. (2019). Impact of a Pre-Operative Exercise Intervention on Breast Cancer Proliferation and Gene Expression: Results from the Pre-Operative Health and Body (PreHAB) Study. *Clin Cancer Res* 25**,** 5398-5406.

Ligibel, J.A., Giobbie-Hurder, A., Olenczuk, D., Campbell, N., Salinardi, T., Winer, E.P., and Mantzoros, C.S. (2009). Impact of a mixed strength and endurance exercise intervention on levels of adiponectin, high molecular weight adiponectin and leptin in breast cancer survivors. *Cancer Causes Control* 20**,** 1523-1528.

Lopes, W.A., Leite, N., da Silva, L.R., Brunelli, D.T., Gáspari, A.F., Radominski, R.B., Chacon-Mikahil, M.P., and Cavaglieri, C.R. (2016). Effects of 12 weeks of combined training without caloric restriction on inflammatory markers in overweight girls. *J Sports Sci* 34**,** 1902-1912.

Nono Nankam, P.A., Mendham, A.E., De Smidt, M.F., Keswell, D., Olsson, T., Blüher, M., and Goedecke, J.H. (2020). Changes in systemic and subcutaneous adipose tissue inflammation and oxidative stress in response to exercise training in obese black African women. *J Physiol* 598**,** 503-515.

Nunes, P.R., Barcelos, L.C., Oliveira, A.A., Furlanetto Júnior, R., Martins, F.M., Orsatti, C.L., Resende, E.A., and Orsatti, F.L. (2016). Effect of resistance training on muscular strength and indicators of abdominal adiposity, metabolic risk, and inflammation in postmenopausal women: controlled and randomized clinical trial of efficacy of training volume. *Age (Dordr)* 38**,** 40.

Olson, T.P., Dengel, D.R., Leon, A.S., and Schmitz, K.H. (2007). Changes in inflammatory biomarkers following one-year of moderate resistance training in overweight women. *Int J Obes (Lond)* 31**,** 996-1003.

Ordoñez, F.J., Fornieles-Gonzalez, G., Camacho, A., Rosety, M.A., Rosety, I., Diaz, A.J., and Rosety-Rodriguez, M. (2013). Anti-inflammatory effect of exercise, via reduced leptin levels, in obese women with Down syndrome. *Int J Sport Nutr Exerc Metab* 23**,** 239-244.

Phillips, M.D., Patrizi, R.M., Cheek, D.J., Wooten, J.S., Barbee, J.J., and Mitchell, J.B. (2012). Resistance training reduces subclinical inflammation in obese, postmenopausal women. *Med Sci Sports Exerc* 44**,** 2099-2110.

Racil, G., Coquart, J.B., Elmontassar, W., Haddad, M., Goebel, R., Chaouachi, A., Amri, M., and Chamari, K. (2016). Greater effects of high- compared with moderate-intensity interval training on cardio-metabolic variables, blood leptin concentration and ratings of perceived exertion in obese adolescent females. *Biol Sport* 33**,** 145-152.

Rogers, L.Q., Fogleman, A., Trammell, R., Hopkins-Price, P., Vicari, S., Rao, K., Edson, B., Verhulst, S., Courneya, K.S., and Hoelzer, K. (2013). Effects of a physical activity behavior change intervention on inflammation and related health outcomes in breast cancer survivors: pilot randomized trial. *Integr Cancer Ther* 12**,** 323-335.

Saeidi, A., Jabbour, G., Ahmadian, M., Abbassi-Daloii, A., Malekian, F., Hackney, A.C., Saedmocheshi, S., Basati, G., Ben Abderrahman, A., and Zouhal, H. (2019). Independent and Combined Effects of Antioxidant Supplementation and Circuit Resistance Training on Selected Adipokines in Postmenopausal Women. *Front Physiol* 10**,** 484.

Soltani, N., Esmaeil, N., Marandi, S., Hovsepian, V., Momen, T., and Shahsanai, A. (2023). A 2-week combined high-intensity interval training regulates inflammatory status in young females with obesity. *Science & Sports* 38**,** 174-181.

Soltani, N., Marandi, S.M., Kazemi, M., and Esmaeil, N. (2020). Meta-inflammatory state and insulin resistance can improve after 10 weeks of combined all-extremity high-intensity interval training in sedentary overweight/obese females: a quasi-experimental study. *J Diabetes Metab Disord* 19**,** 717-726.

Wang, X., You, T., Murphy, K., Lyles, M.F., and Nicklas, B.J. (2015). Addition of Exercise Increases Plasma Adiponectin and Release from Adipose Tissue. *Med Sci Sports Exerc* 47**,** 2450-2455.

Wiklund, P., Alen, M., Munukka, E., Cheng, S.M., Yu, B., Pekkala, S., and Cheng, S. (2014). Metabolic response to 6-week aerobic exercise training and dieting in previously sedentary overweight and obese pre-menopausal women: a randomized trial. *Journal of Sport and Health Science* 3**,** 217-224.

Winters-Stone, K.M., Wood, L.J., Stoyles, S., and Dieckmann, N.F. (2018). The Effects of Resistance Exercise on Biomarkers of Breast Cancer Prognosis: A Pooled Analysis of Three Randomized Trials. *Cancer Epidemiol Biomarkers Prev* 27**,** 146-153.
